# Self‐reported sleep disturbances and its determinants in people 1 year or more after stroke: A cross‐sectional study

**DOI:** 10.1002/pmrj.13329

**Published:** 2025-02-24

**Authors:** Hanna M. Nilsson, Maria Kähler, Lina Rosengren, Lars Jacobsson, Jan Lexell

**Affiliations:** ^1^ Department of Health Sciences, Rehabilitation Medicine Research Group Lund University Lund Sweden; ^2^ Department of Rehabilitation Sunderby Hospital, Norrbotten County Luleå Sweden; ^3^ Department of Rehabilitation Ängelholm Hospital, Skåne County Ängelholm Sweden; ^4^ Department of Health, Education and Technology Luleå University of Technology Luleå Sweden

## Abstract

**Background:**

Survivors of stroke commonly report sleep disturbances. Studies of sleep disturbances after stroke are mostly performed in the acute phase. An increased knowledge of sleep disturbances and its determinants a longer time after stroke is needed to improve treatment and rehabilitation.

**Objective:**

To assess survivors of stroke more than 1 year after stroke onset and (1) investigate self‐reported sleep disturbances and (2) explore the association between self‐reported sleep disturbances, gender, age, time since stroke, other stroke characteristics, and sociodemographic data.

**Design:**

Cross‐sectional survey.

**Setting:**

Community setting.

**Participants:**

Participants (*n* = 160) in the Life After Stroke In Northern Sweden Study (LASINS) (46% women, mean age 73 years, mean time since stroke 35 months).

**Interventions:**

Not applicable.

**Main Outcome Measurements:**

Pittsburgh Sleep Quality Index (PSQI), stroke characteristics (time since stroke, first time stroke, type of stroke, location of stroke, stroke treatment and comorbidities) and sociodemographic data (gender, age, marital status, vocational situation, need for home help, and use of mobility devices).

**Results:**

A total of 84 participants (53%) rated 6 points or more on the PSQI (mean 6.5 points, SD: ±4.2, min‐max 0–18), indicating sleep disturbances. Gender (*p* = .002) and use of mobility devices (*p* = .036) explained 9.5% of the variance in PSQI.

**Conclusion:**

Survivors of stroke report sleep disturbances even several years after stroke onset. Women and those using mobility devices, indicating less recovery after stroke, report sleep disturbances to a higher degree, regardless of chronological age, time since stroke onset, other stroke characteristics, comorbidities, and sociodemographic data. Further studies with a longitudinal design are needed to gain a comprehensive understanding of how stroke‐related factors and other reasons account for poststroke sleep disturbances in order to improve treatment and rehabilitation.

## INTRODUCTION

Stroke is one of the leading causes of death worldwide and is also the disease that most commonly leads to disability in the adult population.^1^, ^2^ People after stroke can experience various impairments that can affect daily living and life satisfaction.^2^, ^3^ One of several impairments after stroke is sleep disturbances.^4^ A good quality sleep is a well‐known factor in achieving physical and mental health and well‐being, among people in general and specifically people with a disability.^5^ To identify and target interventions that optimize sleep among survivors of stroke, we need to investigate the occurrence of sleep disturbances after stroke and explore its determinants.

Several studies have shown that people who have sustained a stroke are more likely to report sleep disturbances than nondisabled people.^6^, ^7^, ^8^, ^9^ Between one third and two thirds of people have reported sleep disturbances in the acute phase after a stroke.^6^, ^7^, ^10^, ^11^, ^12^ Common sleep disturbances that can occur or worsen after stroke are difficulty falling asleep, repeated awakenings during the night, and an increased need for sleep.^13^, ^14^ People who have sustained a stroke also sleep more hours per day, experience sleepiness for three quarters of their waking hours, and are more physically inactive.^15^ A large majority of these studies were pursued during the first year after stroke onset. Thus, knowledge of sleep disturbances in a longer perspective and the association with different stroke characteristics is limited.

Being a woman seems to increase the risk for sleep disturbances in the early phase after stroke.^14^ However, gender differences vary between studies. In a study by Sawadogo et al.,^7^ women were more likely to report sleep disturbances after stroke, whereas Iddagoda et al.^11^ and Jeffers et al.^6^ found no difference between men and women, and Matas et al.^16^ reported that more men than women were bothered by sleep disturbances. Whether chronological age is associated with sleep disturbances after stroke also differs between studies. A meta‐analysis by Baylan et al.^10^ showed that older age is a risk factor for increased sleep disturbances after stroke. However, Iddagoda et al.,^11^ Silva et al.,^17^ and Jeffers et al.^6^ did not show any differences with age. Other sociodemographic data that may be associated with sleep disturbances have been investigated in only a few studies.

Taken together, studies of sleep disturbances after stroke are scarce and mostly performed in the early phase after stroke onset. To the best of our knowledge, there are very few studies on self‐reported sleep disturbances 1 year or more after stroke, and there are inconsistent results regarding the association with gender and age. Moreover, because of cultural and contextual differences, it is challenging to relate findings across studies and different national contexts. Thus, further studies are needed to gain an understanding of poststroke sleep disturbances and its determinants in order to improve treatment and rehabilitation.

The aims of this study are to assess survivors of stroke more than 1 year after stroke onset and (1) investigate self‐reported sleep disturbances and (2) explore the association between self‐reported sleep disturbances, gender, age, time since stroke, other stroke characteristics, comorbidities, and sociodemographic data.

## MATERIALS AND METHODS

### Study design

This study is part of a larger study, the Life After Stroke In Northern Sweden Study (LASINS).^18^ LASINS aims to deepen our understanding of factors important for a healthy and successful life after stroke. The project has a combined quantitative exploratory and descriptive design with a rehabilitation medicine approach including former patients with stroke admitted to a regional hospital in Norrbotten county, northern Sweden. Other studies in LASINS include physical activity, depressive symptoms, fatigue, overall disability, and life satisfaction. Full details of the LASINS with all assessment tools and their psychometric properties can be found in our previous study.^18^ In the present study, a subset of the data from LASINS is used, including the participants' self‐reported sleep disturbances, stroke characteristics, comorbidities, and sociodemographic data. LASINS follows the guidelines of the STrenghtening the Reporting of Observational studies in Epidemiology (STROBE) and contains the necessary items according to the STROBE checklist.^19^, ^20^

### Ethical considerations

All participants received written information about the study, were informed that they could withdraw at any time without giving any reason, and signed an informed consent prior to the start. The LASINS was approved by the Swedish Ethical Review Authority (April 25, 2021; 2021‐01408) and follows the principles of the Helsinki Declaration on research involving humans.

### Study population

The participants in LASINS are all resident in Norrbotten county, the northernmost part of Sweden. Persons who met the following inclusion criteria were invited to participate: diagnosed with stroke (International Statistical Classification of Diseases and Related Health Problems, 10th Revision: I61 cerebral hemorrhage; I63 cerebral infarction; I64 acute cerebrovascular disease not specified as hemorrhage or infarction), treated in the stroke unit at a regional hospital in Norrbotten County between the years 2017 and 2019, living in their own home, over 18 years old and able to understand and answer written self‐assessment tools and questionnaires in Swedish. Exclusion criteria in the LASINS were severe cognitive impairment (such as dementia), moved abroad, and diagnosed with a transient ischemic attack (TIA).

Potential participants were identified through review of medical records. A total of 1518 persons had been treated for stroke at the hospital during the 3‐year period. Of these, 949 persons had been treated in the stroke unit, and of those 301 met the inclusion criteria and were invited to participate; the remaining were deceased (*n* = 245) or did not meet the inclusion criteria (*n* = 403; eg, TIA or living in a nursing home). A total of 160 people accepted the invitation (response rate 53%) and comprised the final sample.^18^

### Data collection procedures

All potential participants (*n* = 301) were mailed information about the study, an informed consent form, a questionnaire with sociodemographic data, the self‐assessment tools, and a prestamped and addressed reply envelope. One reminder was sent out after the first invitation.

### Data obtained from the medical records and sociodemographic questionnaire

Data about stroke characteristics (time since stroke onset, first‐time stroke, type of stroke, location of the stroke, stroke treatment [ie, thrombolysis and/or thrombectomy], and comorbidities) were obtained from the participants' medical records. The sociodemographic questionnaire included: gender (man or woman), age, marital status, vocational situation, need of home help, and use of mobility devices.

### Self‐reported sleep disturbances

The Pittsburgh Sleep Quality Index (PSQI) was used to assess sleep disturbances among the participants. The PSQI was developed to provide a reliable and standardized self‐report assessment of sleep quality, to distinguish good sleepers from poor sleepers, and to provide a tool that is easy to use for persons and easy to interpret for clinicians and researchers.^21^

The PSQI asks about sleep during the past month and contains several questions that are divided into seven components of which each assesses a particular clinical aspect of sleep: (1) subjective sleep quality, (2) sleep latency, (3) sleep duration, (4) habitual efficiency, (5) sleep disturbances, (6) use of sleeping medications, and (7) daytime dysfunction. Each of the seven components yields a score between 0 and 3 where 0 is no difficulties and 3 is severe difficulties. These scores are then summed to provide a global score from 0 to 21. A global score of 6 points or more has been found to yield a diagnostic sensitivity of 89.6% and specificity of 86.5% in distinguishing good sleepers from poor sleepers.^21^

PSQI can be used for different populations.^22^ It takes between 5 and 10 minutes to complete and about 5 minutes to calculate the global score.^21^, ^23^ The PSQI has a correlation of 0.80 with other questionnaires and an intraclass correlation coefficient from 0.70 to 0.86,^23^ indicating good validity and reliability.^24^ It also has good internal consistency with a Cronbach's alpha from 0.70 to 0.83.^21^, ^23^

### Statistical analysis

Data are presented using descriptive statistics with mean, median, SD, minimum, maximum, frequency and proportion (%), where appropriate.

To investigate the association between PSQI, gender, age, time since stroke, other stroke characteristics (first‐time stroke, type of stroke, location of stroke, and stroke treatment), comorbidities and sociodemographic data (marital status, vocational situation, need of home help, and use of mobility devices), a hierarchical multiple regression analysis was conducted with PSQI as the dependent variable. The independent variables included were gender, age, time since stroke, other stroke characteristics, comorbidities, and sociodemographic data. The variables type of stroke, vocational situation, need of home help, and use of mobility devices had three or four response options and were transformed into bivariate variables. Dummy variables were created for location of stroke with the left hemisphere as the reference category.

The hierarchical regression analysis was conducted in three steps. Gender and age were included in the first step; time since stroke, other stroke characteristics, and comorbidities were included in the second step; and in the third and final step sociodemographic data were included. There was no bivariate correlation above 0.56, the variance inflation factor for all variables was <2.1 and the tolerance was ≥0.48; thus, there were no indications of multicollinearity. A residual analysis was also conducted to confirm the validity of the regression model.^25^ The F‐statistics was used to compare model performance between the different hierarchical models.

Throughout, *p* values <.05 were considered statistically significant. All statistical analyses were performed using IBM SPSS Statistics Software v 28 (IBM Corporation, Armonk, NY, USA).

## RESULTS

### Participant characteristics

In Table 1, the characteristics of the participants are presented. Of the 160 participants, 86 were men (54%) and 74 were women (46%), and the mean age was 73 years (SD: ±11; 30–91). The mean time since stroke onset was 35 months (SD: ±11; range 18–61) and 139 (87%) had sustained an ischemic stroke. Most participants (*n* = 112; 70%) had a hemispheric stroke, 36 (23%) had received treatment with thrombolysis and/or thrombectomy, and most of the participants had a first‐time stroke (*n* = 136; 85%). A large majority had some kind of comorbidity (*n* = 143; 89%) with hypertension, atrial fibrillation, hyperlipidemia, and type 2 diabetes being the most frequently reported. The majority of the participants were retired (*n* = 131; 82%) and two thirds were cohabiting (*n* = 100; 62.5%). Most participants did not need any home help (*n* = 134; 84%) and the majority did not use any mobility devices (*n* = 113; 71%).

**TABLE 1:** Characteristics of the 160 participants.

Gender	
Men	86 (54)
Women	74 (46)
Age (years)	73 ± 11; 74, 30–91
Time since stroke (months)	35 ± 11; 34, 18–61
First‐time stroke	136 (85)
Type of stroke	
Ischemia	139 (87)
Subarachnoid hemorrhage	0
Intracerebral hemorrhage	21 (13)
Location of stroke	
Right hemisphere	53 (33)
Left hemisphere	59 (37)
Cerebellum	13 (8)
Other^a^	35 (22)
Stroke treatment	
Thrombolysis	33 (21)
Thrombectomy	3 (2)
Comorbidities^b^	143 (89)
Marital status	
Living alone	60 (37.5)
Cohabitant	100 (62.5)
Vocational situation	
Working	26 (16)
Not working	134 (84)
Need of home help	26 (16)
Use of mobility devices	47 (29)

*Note*: Data are presented as *n* (%) and mean ± SD; median, min‐max.

^a^ Other = stroke localized in the brainstem, both hemispheres or unidentified.

^b^ The most frequently reported were hypertension, atrial fibrillation, hyperlipidemia, and type 2 diabetes.

### Self‐reported sleep disturbances

**FIGURE 1:**
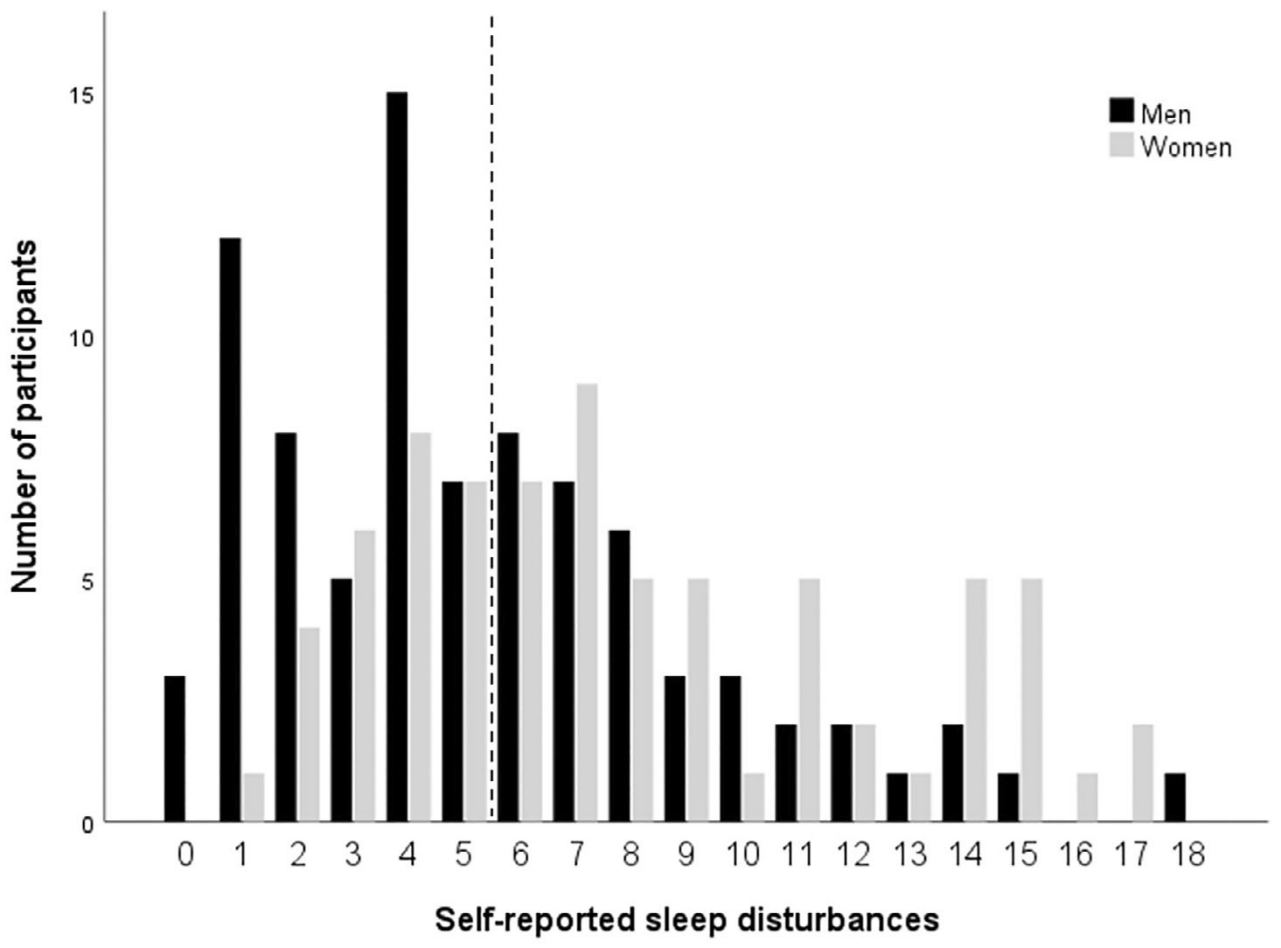
Distribution of self‐reported sleep disturbances among the 160 participants (86 men and 74 women) assessed with Pittsburgh Sleep Quality Index (PSQI). The vertical dashed line represents the cutoff value. Six points or more indicate sleep disturbances and higher scores indicate more sleep disturbances.

In Figure 1, the distribution of self‐reported sleep disturbances for the 86 men and the 74 women are presented. The mean score of the total PSQI was 6.5 (SD: ±4.2; min–max 0–18) and the median 6 (q1–q3 4–9). For the men the mean PSQI score was 5.4 (SD: ±3.8; min–max 0–18) and the median 4.5 (q1–q3 2–7), and for the women the mean PSQI score was 7.9 (SD: ±4.3; min–max 1–17) and the median 7 (q1–q3 4–11). Of the 160 participants, 84 (53%) scored 6 points or more on the PSQI; of those, 36 (43%) were men and 48 (57%) women. The majority of the participants scored around 5 points, a few participants scored 16 points or more, and no participant scored more than 18 (of 21) points.

### The association between sleep disturbances, gender, age, time since stroke, other stroke characteristics, and sociodemographic data

In Table 2, the results from the hierarchical regression analysis with PSQI as the dependent variable are presented. In the first step, with gender and age as independent variables, the model was significant and explained 7.6% (*p* < .001) of the variance in PSQI, with gender as a single significant contributor (*p* < .001). In the second step, stroke characteristics and comorbidities were included. This step did not result in a significant change in the model; the model explained 8.2% of the variance in PSQI, and with gender as a single contributor. The third and final step included sociodemographic data. The change in this model was not significant and the model explained 9.5% of the variance in PSQI; both gender (*p* = .002) and use of mobility devices (*p* = .036) contributed significantly to the variance in PSQI.

**TABLE 2:** Results of the hierarchical regression analysis for the 160 participants with Pittsburgh Sleep Quality Index (PSQI) as dependent variable.

PSQI			
	Step 1	Step 2	Step 3
Gender	0.291 (<**.001**)	0.269 (<**.001**)	0.250 (**.002**)
Age	−0.045	−0.062	−0.051
Time since stroke		0.096	0.063
First‐time stroke		0.033	0.015
Type of stroke		−0.029	−0.057
Location of stroke^a^			
Right hemisphere		−0.077	−0.051
Cerebellum/other^b^		−0.174	−0.164
Stroke treatment		0.010	−0.023
Comorbidities		−0.103	−0.074
Marital status			−0.063
Vocation			−0.109
Need of home help			−0.086
Use of mobility devices			0.206 (**.036**)
Significance	<0.001	0.009	0.009
R^2^ adj	0.076	0.082	0.095
R^2^ change	0.088	0.047	0.035
F change	7.500	1.150	1.514
F ratio	7.500	2.572	2.271
Sig F change	<0.001	0.335	0.201

*Note*: Standardized beta coefficients are presented. Bold values in parentheses represent significant *p* values (*p* < .05).

^a^ Location of stroke was transformed to dummy variables with left side stroke as the reference category.

^b^ Other = stroke localized in the cerebellum, brainstem, both hemispheres, and unidentified.

## DISCUSSION

Increased knowledge about poststroke sleep disturbances and its determinants is needed to improve treatment and rehabilitation for survivors of stroke. The main finding in this study was that 53% of the participants reported 6 points or more on PSQI more than 1 year after stroke, indicating sleep disturbances. Being a woman and using mobility devices explained 9.5% of the variance in sleep disturbances.

A majority of the participants in the present study reported sleep disturbances, which is in agreement with previous studies, both in a short‐term and long‐term perspective after stroke. In a meta‐analysis including 14 studies with a total sample size of 16,894 survivors of stroke, up to 69% reported sleep disturbances 3 days to 18 months after stroke onset.^10^ In a register study from Canada including 682 survivors of stroke and 45,722 controls, 61.6% reported some kind of sleep disturbances after stroke, which was higher than in the control group.^6^ In a study from Korea, including 145 participants, 54% reported sleep disturbances 40 to 51 months after stroke onset.^26^

Most previous studies have used PSQI to assess sleep disturbances.^10^, ^11^, ^12^, ^26^ Few studies have used other validated and nonvalidated self‐assessment tools.^6^, ^7^, ^10^ As noted, the number of participants differs greatly between studies. The majority of previous studies are of similar size as the present study, whereas studies using existing registers are based on a larger sample size.^6^, ^7^

When comparing the prevalence of self‐reported poststroke sleep disturbances with a healthy population, about 10% of a healthy population are bothered by sleep disturbances and up to 30% by at least one type of sleep disturbance.^27^, ^28^, ^29^, ^30^ Despite differences in the choice of assessment tools, number of participants, geographical location, and choice of research methods, it appears that survivors of stroke report sleep disturbances to a higher degree than the general population.

In the present study, women reported significantly more sleep disturbances than men. In previous studies there was a discrepancy regarding gender and sleep disturbances after stroke. Some studies reported no difference between men and women,^6^, ^11^ whereas other studies showed a gender difference.^7^, ^14^, ^16^ In general, women report a more reduced sleep quality than men, regardless of disease or not.^31^, ^32^, ^33^ The mechanisms underlying this gender difference is not fully understood, and further studies are needed to elucidate the difference between men and women regarding sleep disturbances after stroke.

The use of mobility devices was also associated with sleep disturbances in the present study. Needing a mobility device implies that the person is more severely affected by stroke and less recovered.^34^ This is in line with previous studies that have shown that those who have physical functional impairments after a stroke to a higher degree report sleep disturbances.^35^ Thus, the results in this study support the contention that sleep disturbances are reported to a higher degree in people who are more severely affected by a stroke.

In the present study, the participants reported sleep disturbances regardless of first‐time stroke, type of stroke (infarction or hemorrhage), location of stroke, whether they had received thrombolysis and/or thrombectomy, and comorbidities. This is also in agreement with the few other studies that have included these factors.^10^

In terms of time since stroke onset, there was no difference in self‐reported sleep disturbances regardless of when the participants had their stroke. This implies that poststroke sleep disturbances can persist over several years, which has also been shown in previous studies.^6^, ^10^, ^26^ Generally, though, the time aspect after stroke has received much less interest in the past. Few studies have been conducted a longer time after stroke onset, and the time aspect has usually only been defined as “after stroke” or “with a history of stroke,” but not more specifically.^6^, ^7^, ^10^ According to Bakken et al.,^14^ Leppävouri et al.,^13^ and Iddagoda et al.,^11^ it appears that sleep disturbances after stroke improve between 1 month and up to 1 year after the stroke. The results of the present study cannot, due to its design, conclude whether improvements in sleep have occurred among the participants, but the results indicate that self‐reported sleep disturbances after a stroke can remain up to more than 5 years.

Age was not significantly associated with self‐reported sleep disturbances in the present study. On one hand, Iddagoda et al.^11^ and Silva et al.^17^ also found no association between sleep disturbances after stroke and age, regardless of the age of the participants. On the other hand, Kim et al.,^36^ Palomäki et al.^37^ and Bakken et al.^14^ showed that older age was associated with self‐reported sleep disturbances after stroke; in their studies the mean age varied between 55 and 70 years. Further studies are needed to elucidate if higher age is associated with more sleep disturbances.

Finally, there was no significant association between self‐reported sleep disturbances and the remaining sociodemographic data, which suggests that sleep problems can occur regardless of marital status, vocational situation, and need of home help.

### Clinical implications and future research

Due to the importance of sleep for health and quality of life, systematic screening for sleep disturbances among people after stroke is advocated, from the acute phase to the long‐term follow‐up. Targeted interventions for sleep disturbances are likely to improve poststroke rehabilitation and individual outcomes, leading to an improved quality of life for survivors of stroke. Further studies are needed to elucidate the underlying mechanisms of poststroke sleep disturbances and find the most effective interventions to manage sleep disturbances after stroke.

Based on previous research, it is known that sleep disturbances are associated with other factors, such as impaired physical and cognitive function.^3^, ^9^, ^38^ There is also a link between sleep disturbances, depressive symptoms, and fatigue after stroke.^38^, ^39^ However, very few studies explore these associations in a long‐term perspective. Thus, to gain a more comprehensive knowledge in this area there is a need for more research on sleep disturbances a longer time after stroke onset and the association with other factors.

### Strengths and limitations

One strength of this study is the time perspective since stroke onset. Also, the participants in this study have been considered to represent the global stroke population and are representative of the stroke population in northern Sweden.^18^ The chosen self‐assessment tool—PSQI—is internationally validated, well used and reliable to assess self‐reported sleep quality. There are also some limitations. This study used a relatively small geographical area for selection of participants with only one hospital included and had a relatively low response rate, which may limit the generalization of the results. Furthermore, we did not account for sleep disturbances before stroke onset and to what extent the participants' sleep disturbances could be due to the stroke itself or other factors. Also, we did not record all possible comorbidities that may influence sleep, for example sleep apnea and nocturia. Given the cross‐sectional design of the study, we cannot make conclusions about the causal inferences or whether there has been any change in sleep quality over time on an individual level. Therefore, future studies need to have a prospective and longitudinal design including potential comorbidities that could influence sleep. Finally, the participants are relatively recovered after their stroke,^18^ so the results cannot be generalized to people with more severe consequences of stroke.

## CONCLUSIONS

Survivors of stroke report sleep disturbances even several years after stroke onset. Women and those using mobility devices, indicating less recovery after stroke, report sleep disturbances to a higher degree, regardless of chronological age, time since stroke onset, other stroke characteristics, comorbidities, and sociodemographic data. Further studies with a longitudinal design are needed to gain a comprehensive understanding of how stroke‐related factors and other reasons account for poststroke sleep disturbances in order to improve treatment and rehabilitation.

## AUTHOR CONTRIBUTIONS

HMN, MK, LR, LJ, and JL conceptualized the study. HMN and MK performed the data collection and analysis. HMN and JL drafted the original version of the manuscript. All authors reviewed and edited the revised versions and subsequently approved the final version of the manuscript.

## DISCLOSURES

The authors declare that there is no conflict of interest to declare.

## Data Availability

All data are archived according to the Swedish Act concerning the Ethical Review of Research Involving Humans and are available from the authors upon request.
